# Qualitative systematic reviews of treatment burden in stroke, heart failure and diabetes - Methodological challenges and solutions

**DOI:** 10.1186/1471-2288-13-10

**Published:** 2013-01-28

**Authors:** Katie Gallacher, Bhautesh Jani, Deborah Morrison, Sara Macdonald, David Blane, Patricia Erwin, Carl R May, Victor M Montori, David T Eton, Fiona Smith, David G Batty, Frances S Mair

**Affiliations:** 1 University of Glasgow, Scotland, UK; 2 Mayo Clinic, Rochester, MN, USA; 3 University of Southampton, England, UK; 4 University College London, England, UK

**Keywords:** Qualitative systematic review, Normalization process theory, Stroke, Treatment burden

## Abstract

**Background:**

Treatment burden can be defined as the self-care practices that patients with chronic illness must perform to respond to the requirements of their healthcare providers, as well as the impact that these practices have on patient functioning and well being. Increasing levels of treatment burden may lead to suboptimal adherence and negative outcomes. Systematic review of the qualitative literature is a useful method for exploring the patient experience of care, in this case the experience of treatment burden. There is no consensus on methods for qualitative systematic review. This paper describes the methodology used for qualitative systematic reviews of the treatment burdens identified in three different common chronic conditions, using stroke as our exemplar.

**Methods:**

Qualitative studies in peer reviewed journals seeking to understand the patient experience of stroke management were sought. Limitations of English language and year of publication 2000 onwards were set. An exhaustive search strategy was employed, consisting of a scoping search, database searches (Scopus, CINAHL, Embase, Medline & PsycINFO) and reference, footnote and citation searching. Papers were screened, data extracted, quality appraised and analysed by two individuals, with a third party for disagreements. Data analysis was carried out using a coding framework underpinned by Normalization Process Theory (NPT).

**Results:**

A total of 4364 papers were identified, 54 were included in the review. Of these, 51 (94%) were retrieved from our database search. Methodological issues included: creating an appropriate search strategy; investigating a topic not previously conceptualised; sorting through irrelevant data within papers; the quality appraisal of qualitative research; and the use of NPT as a novel method of data analysis, shown to be a useful method for the purposes of this review.

**Conclusion:**

The creation of our search strategy may be of particular interest to other researchers carrying out synthesis of qualitative studies. Importantly, the successful use of NPT to inform a coding frame for data analysis involving qualitative data that describes processes relating to self management highlights the potential of a new method for analyses of qualitative data within systematic reviews.

## Background

### Treatment burden

Recently, there has been a growing literature that describes the concept of *treatment burden.* Treatment burden can be defined as the “workload” of health care that patients must perform in response to the requirements of their healthcare providers as well as the “impact” that these practices have on patient functioning and well being. “Workload” includes the demands made on a patient’s time and energy due to treatment for a condition(s) (e.g. attending appointments, undergoing investigations, taking medications) as well as other aspects of self-care (e.g. health monitoring, diet, exercise). “Impact” includes the effect of the workload on the patient’s behavioural, cognitive, physical, and psychosocial well-being [1,2]. Two patients with equivalent “workloads” may be burdened in different ways and to different extents, this can be explained by differences in their “capacity”, meaning their ability to handle work (e.g. functional morbidity, financial/social resources, literacy) as well as the burden of the illness itself [2]. It has been posited that treatment burden is important because for many people with complex, chronic co-morbidities it may reduce their capacity to follow management plans [3]. Those individuals with chronic illness who view their management plans as being excessively demanding are less likely to adhere to therapies [4,5]. Thus, increasing treatment burden, which is more likely in those with multiple chronic conditions, may lead to suboptimal adherence and consequently negative outcomes [3]. This can lead to further burden of illness and more intensified treatments, further increasing the burden on the patient. Treatment burden is therefore part of a dynamic state involving a complex set of personal, social and clinical factors contributing towards the patient’s experience [2].

A range of treatment burdens or workload factors for those with chronic disease have been described which include: *logistical burdens*, for example organising appointments or visits from health professionals, organising rehabilitation, arranging transport; *technical burdens*, for example enacting lifestyle changes, performing rehabilitation exercises, modifying environments, taking medications*; relational burdens*, for example enrolling family, friends and health professionals for support, initiating interactions with possible carers and supporters; and *sense making burdens*, for example conceptualising problems, understanding and learning about management strategies, knowing when to seek help, differentiating between treatments [6-9].

Although aspects of treatment burden have been described we do not yet have a full understanding of the phenomenon, and in particular, what might be the generic or disease specific features. Our aim is to explore treatment burden as a concept, with the aim of informing the development of a method of measurement [10], in order to aid clinicians and policy makers in decreasing treatment burden for patients [11]. It is for this reason that we have conducted three systematic reviews of the qualitative literature relating to patient experiences of living with stroke, heart failure and diabetes. These three chronic diseases were chosen as we hypothesised that they all involve conceivably complicated, long term management plans that require significant personal investment from patients [12-14].

### Systematic review of qualitative studies

We chose to examine the qualitative literature as this type of research suitably lends itself to uncovering and exploring patients’ perceived needs and behaviours, providing conceptual depth about the patient experience. However, conducting a qualitative systematic review remains challenging and contentious. Increasing numbers of qualitative studies have led to a demand for reliable methods for appraising and synthesising qualitative research similar to the systematic review and meta-analysis of quantitative studies [15,16]. However, there are opposing views on whether this is appropriate or even possible, due to deep seated epistemological and ontological differences [17].

There are a range of methods available for the synthesis of qualitative research [18]. With regards to searching the literature, there are two main schools of thought: those who advocate using purposeful sampling to retrieve materials until data saturation is reached [19]; and those who aim to retrieve all of the relevant studies in a field rather than a sample of them [20]. The first approach is often taken by authors of narrative reviews, reviews using an extremely large and diverse set of resources [21], or those aimed at developing concepts and theories rather than summarizing research carried out to date [22]. Studies aimed at comprehensively summarizing the literature should include a comprehensive and rigorous database search using predefined index/subject heading/free text terms, informed by an initial scoping search [22-26].

Finding relevant qualitative studies has been reported as an arduous task due to inadequate refinement of the electronic indexing of qualitative articles [22,27]. Those papers found in journals also often lack abstracts or include titles based on patient quotes, making it difficult to establish relevance of the paper in question [22]. Several papers have been published outlining strategies for searching through well known databases for relevant qualitative studies [28-31].

Due to these difficulties, other techniques have become established as helpful in the searching process, which can involve either electronic or hand searching [25,26]:

– Reference or footnote tracking (looking back at studies referenced in articles found).

– Citation tracking (looking forward at studies that have subsequently cited articles found, using a citation database).

– Personal knowledge and personal contacts.

– Contacting the authors of known papers or experts in the field.

– Hand searching relevant journals.

– Internet browsing such as berry picking (a method of searching where one search may lead to another and ‘clusters’ of papers are often found together).

Indeed, in their systematic review of complex evidence, Greenhalgh et al. found that only 30% of their primary sources were found by the traditional method of using a predefined search strategy and that 51% were found by other predefined methods such as reference, footnote and citation tracking [25].

There are opposing thoughts on whether quality appraisal of qualitative research is appropriate. Those against it believe that each piece of research tells its own story and cannot be compared to another [16]. Others, however, believe it to be an essential component of rigorous qualitative synthesis [15], albeit amongst these supporters there is no consensus on how to enact quality appraisal, unlike the widely agreed checklists available for quantitative research, such as the Cochrane Risk of Bias Tool [32-34].

Methods of data synthesis are also highly debated, with a great array of documented options and somewhat confusing terminology [18]. Most consist of a ‘compare and contrast’ exercise, which can range from descriptive techniques that aim to summarize similarities and differences between studies and interpretive techniques that additionally aim to develop new understandings and perspectives while preserving meaning from the original studies [23,24]. Examples of techniques used include meta-ethnography [35], critical interpretive synthesis [36], thematic synthesis [37], grounded theory [38], meta-narrative review [39], realist synthesis [21], cross case analysis [40], meta-synthesis [41], and meta-study [42]. Meta-ethnography has emerged as one of the more popular methods of data synthesis [27,35]. This is an interpretive method that seeks to create higher order interpretations, and tends to be suited to researchers using inductive methods of research seeking to explore a phenomenon rather than answer a predefined question [18].

It has been suggested that Normalization Process Theory (NPT) [43,44] could potentially offer new ways to approach the analysis of qualitative data gathered as part of a systematic review and that it could have a role in helping to interpret data when considering how patients or carers manage/deal with a range of conditions and self care issues [45]. NPT has a robust theoretical basis and explains how the work of enacting an ensemble of practices (in this case the components of treatment burden) is accomplished through the operation of four mechanisms: ‘coherence’ (sense making work); ‘cognitive participation’ (relationship work); ‘collective action’ (enacting work); and ‘reflexive monitoring’ (appraisal work) [43]. NPT has previously been used successfully to aid conceptualisation of the qualitative literature relating to the implementation of new technologies by framework synthesis [45-47]. Framework synthesis is a method of synthesis derived from qualitative framework analysis [48,49]. It is an appropriate method for researchers with some degree of knowledge in their chosen area, with a predefined framework being applied to data to gain a deeper understanding of a particular phenomenon. Care must be taken, however, not to ‘shoe horn’ findings into the framework, and this is one challenge of using such a method. A novel aspect of our reviews is that we have used NPT as a conceptual and coding framework and we describe this approach within this paper.

### Aims

The aim of this paper is to describe and discuss the methods used and instruments developed to undertake qualitative systematic reviews of the treatment burdens identified in three different common chronic conditions. The approaches used for data collection and analysis were the same for all three. A particularly novel aspect of these reviews is the use of a coding framework underpinned by NPT. In this paper we use the stroke review as our exemplar.

## Methods

### Searching for papers

Qualitative studies using techniques involving direct patient contact or observation such as interviews and focus groups, seeking to understand the patient experience of stroke management were sought. An exhaustive search strategy was deemed suitable, as the aim was to summarise the literature on this topic. Limitations of English language and year of publication 2000 and onwards were set. There were no geographical restrictions.

‘Scoping searches’ were carried out with the aim of identifying key papers and familiarising reviewers with key terms. This consisted of: searching our own files; internet searching using the ‘berry picking’ method (a method of searching where one search may lead to another and ‘clusters’ of papers are often found together) [26]; a preliminary search of databases via Ovid; the use of the ‘related articles’ function in Pubmed (http://www.ncbi.nlm.nih.gov/pubmed/) and Web of Science (http://wok.mimas.ac.uk/); and consultation with experts in the field.

A formal database search strategy was created in consultation with an information scientist, informed by key words and phrases found during the scoping search. Additional file 1 shows the full search strategy created using a combination of free text search terms and subject headings. Databases searched were Scopus, CINAHL, Embase, Medline & PsycINFO. The search initially centred around three main concepts: ‘stroke’; ‘treatment burden’; and ‘patient experience’ then the concept ‘qualitative methods’ was added to increase sensitivity and specificity. Reference, footnote and citation tracking were then carried out on included papers. The references were also searched of 10 reviews found during the scoping search that examine the qualitative literature on the patient experience of stroke, none directly aimed at understanding treatment burden, but on related topics.

### Paper screening

Each title, abstract and full paper was screened by two individuals (KG, DM, BJ, SM) with a third party involved for any disagreements (FM). Additional file 2 illustrates inclusion and exclusion criteria used. Inclusion of studies was limited to those that involve direct patient contact or observation such as interviews or focus groups, with qualitative methods of analysis that seek to identify themes or patterns discussed by participants. Studies using telephone, postal or internet questionnaires were excluded, as were those using document analysis, quantitative patient-reported measures, simple counts of patient responses, and language analysis presented as quantitative results. We included studies that explored the patient experience in any setting, but excluded those investigating the patient experience of pilot or experimental studies rather than ‘real world’ settings. This meant that qualitative studies as part of a mixed methods study would be included, but only if these pertain to usual patient care, rather than the patient experience of, for example, an experimental treatment regime.

Studies seeking to understand the patient experience of stroke management with a focus on treatment burden were included. Due to the novel nature of our research question, we found that screening papers consistently was difficult, as treatment burden was not typically the focus of the paper, with relevant information being somewhat ‘hidden’ in the results. We therefore found that screening often came down to a judgment about ‘how relevant’ a paper was. To improve consistency yet be as inclusive as possible we agreed that for inclusion, roughly over 30% of the results and discussion within a paper should focus on treatment burden. Due to the possible subjective nature of this decision, we only excluded papers that two reviewers excluded for the same reason, with any conflicts going to a third party for review.

### Data extraction

Data extraction was conducted by two individuals (KG, DM, BJ, SM) with a third party involved for any disagreements (FM). Data extracted for analysis was limited to data describing a range of treatment burdens. Clear criteria for inclusion and exclusion of data were used to inform decision making as illustrated in Additional file 3. Both researchers screened all data from the results and discussion of every included paper with a third party for disagreements, to ensure inclusion of all relevant data. The data extraction instrument developed and used is shown in Additional file 4. A careful note was made of any treatment burden data that fell outside our framework in order to assess if our framework was ‘fit for purpose’ and to ensure that no relevant data was missed.

### Data analysis

A particularly novel aspect of this review was our approach to data analysis. To facilitate understanding of the components and dimensions of treatment burden, we utilised Normalization Process Theory (NPT).

Data were analysed using a coding frame informed by NPT, following the five stages of framework analysis: familiarisation, identifying a thematic framework, indexing, charting, mapping and interpretation [48]. The framework was underpinned by NPT and informed by a previous study that involved the analysis of semi-structured, qualitative interviews with heart failure patients [6], as well as our knowledge of the literature and clinical experience. It was then adapted and refined during analysis to create a stroke specific coding frame for treatment burden. This was used to identify, describe and understand the components of treatment burden experienced by patients with stroke. The coding frame underpinned by NPT developed for data analysis of the stroke literature is shown in Table 1. Analysis was conducted by two individuals (KG, DM, BJ, SM) with a third party involved for any disagreements (FM). As well as the regular meetings between the two coders, ‘coding clinics’ were held on several occasions, involving a group of six researchers (three of whom have considerable experience in this field) all coding transcripts separately and discussing any differences. Refinement of the coding frame and analysis was therefore iterative.

**Table 1:** NPT based coding framework

**COHERENCE**	**COGNITIVE PARTICIPATION**	**COLLECTIVE ACTION**	**REFLEXIVE MONITORING**
*(Sense-making work)* Understanding the prospect of having, what this means and how the condition may be managed.	*(Relationship work)* Investing personal and interpersonal commitment to living with the condition and its management.	*(Enacting work)* Investing effort and resources in management and carrying out necessary tasks.	*(Appraisal work)* Reflecting on the effects of therapies in retrospect and determining whether to modify them.
**Differentiation**	**Enrollment**	**Skill set workability**	**Reconfiguration**
Understanding and differentiating between risk factors, investigations, treatments and the roles of different health professionals and services. Prioritising treatments and activities.	Engaging with friends, family and health professionals with regards to diagnosis and illness management to enable them to provide support. Adjusting relationships to accommodate new roles as a result of illness during management.	Setting a routine to cope with symptoms, exacerbations, and emergency situations i.e. therapeutic interventions. Enacting activities with a view to achieving goals. Controlling risks associated with recovery.	Altering a set routine when required such as medication regimes or appointments, to fit in with daily activities ot other arrangements. Learning a new way of doing things after sroke. Altering priorities and ways of thinking due to stroke management.
**Communal specification**	**Activation**	**Contextual Integration**	**Communal Appraisal**
Gaining information about illness management with the help of others, for example friends, family or health professionals. Receiving diagnosis, or misdiagnosis.	Arranging help (e.g. logistical, administrative, or expert) from health professionals, social services or friends and family.	Making sure you have the right financial and social resources, and integrating the illness into social circumstances. Managing potential environmental dangers through making resources available. Adjusting to new social role in society or life circumstances such as unemployment.	Discussing or altering current management plans already initiated, in discussion with health professionals or friends and family. Recalling previous events with friends and family.
**Individual specification**	**Initiation**	**International workability**	**Individual appraisal**
Achieving your own understanding of illness management in personal terms, through personal research such as reading, or personal experience.	Using organisational skills to arrange one’s own contributions to management, such as arranging prescriptions, social care and transport to appointments.	Taking treatments, enacting lifestyle changes, attending appointments, enduring side effects. Enduring poor health care or care that does not meet expectations (e.g. poor interactions). Enduring setbacks in recovery. Learning self care. The work of rehab. The work after discharge. Enduring intrusions and interventions from family members, including negative interactions.	Assessing individually whether to continue or alter current management plans. Recalling previous events. Monitoring symptoms and progress (but not as a routine, see below).
**Internalization**	**Legitimation**	**Relational Integration**	**Systematization**
Relating your experience to illness management, understanding any implications, knowing when to seek help, understanding one’s own contributions to reducing risk, knowing limitations and risks due to stroke. Calculating safety risks. Maintaining motivations and determination. Developing expectations of health services. Making sense of progress in recovery and one’s own contributions to this. Setting goals for recovery.	Seeking reassurance from others about appropriateness of management plans. Gaining confidence in the success of treatments. Dealing with stigmatisation or a mismatch in ideas and expectation from others. Reaching an understanding that treatments are ‘the right thing to do’. Comparing yourself to others to validate treatments.	Maintaining confidence in health professionals and their interaction with each other. Maintaining confidence in care plan. Coping with multiple caregivers. Enduring system failures caused by poor communication/interaction by service provides.	Developing ways of keeping up to date with newly available treatments. Routine self monitoring.

All data was coded according to the NPT framework, with data being coded under the four NPT domains (coherence, cognitive participation, collective action, reflexive monitoring) and their subconstructs (see Table 1). Several codes were created within each subconstruct, and these were subsequently grouped together under treatment burden headings. This created a taxonomy of treatment burden that reflects the original accounts of the patients being studied, so could be described as ‘grounded’ in the data, with the framework underpinned by NPT being used for initial extraction and organisation.

### Quality appraisal

A quality appraisal instrument was created and based upon published guidance by well known qualitative researchers [17]. This is shown in Additional file 5. From this guidance, the authors developed an instrument consisting of eleven questions, each considering an aspect of quality such as rigour, validity, transparency and generalisability. Two researchers independently carried out quality appraisal and answers were compared and discussed. No scoring system or level of ‘pass mark’ was set as the value of this is uncertain [50]. Appraisal was therefore not carried out to exclude studies but to inform the discussion and analysis. This involved creating a summary of the quality of included studies, in order to highlight any notable defects in the quality of the literature, as well as to inform our own future qualitative research in this area.

## Results

### Searching and screening

**Figure 1:**
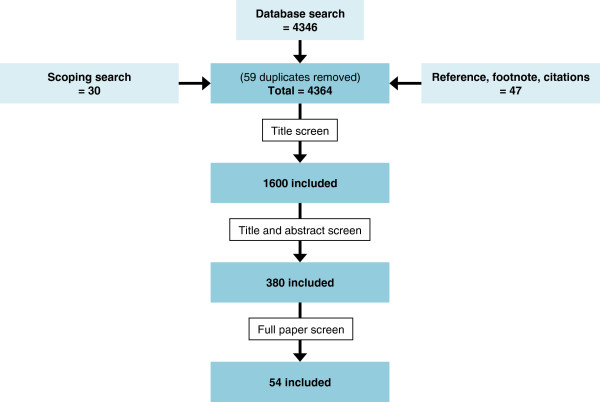
Flowchart demonstrating papers included in the stroke review.

Our scoping search uncovered 10 key papers, 10 reviews and 20 potentially relevant papers. The initial search which centred on the concepts of stroke; treatment burden; and patient experience retrieved over 30,000 papers. This was not deemed adequately specific or sensitive as some key papers were not retrieved. A second search strategy was then created, adding the concept ‘qualitative methods’ [28-31,51,52]. This significantly increased sensitivity and specificity of the search: 4346 papers were identified; all key papers were retrieved. Another 47 papers were identified from reference, footnote and citation tracking of all included papers. At full paper screen level, 33 out of 380 papers required review by a third party due to conflict between the first and second reviewer. 54 papers reached the final stage of data extraction and analysis (see Figure 1). Of these 54 papers, 51 (94%) were retrieved from our database search. The 3 papers included that were not found in our database search were found from reference searching (see Figure 1).

### Data extraction and analysis

Table 1 displays the NPT coding framework used for analysis. No data on treatment burden was found that fell outside this coding framework, an important finding as this provides evidence that NPT is suitable for conceptualising the treatment burden faced by patients with chronic illness. We identified the following areas of treatment burden from the literature*: making sense of treatments* e.g. gaining information from health professionals*; planning recovery and care* e.g. setting goals*; interacting with others* e.g. coping with multiple caregivers; *institutional admissions* e.g. admission to hospital; *managing stroke in the community* e.g. risk factor management at home; *reintegrating into society* e.g. addressing financial difficulties; *adjusting to life after stroke* e.g. planning a new daily structure to accommodate treatments; and *reflecting on management* e.g. making decisions about adherence.

The following examples are excerpts from included papers with a demonstration of how these were coded. See Table 1 for a detailed description of each code. The first is an example of Coherence; Communal Specification (COCS). This describes poor information provision from health professionals to patients, and is categorised in our treatment burden taxonomy as ‘making sense of treatments’:

*Not being adequately informed concerned what the participants described as absent, contradictory or incomprehensible information. Some of them had not received any information other than what was given to them in a brochure about stroke. Others had wanted more detailed information about their brain injury, the reason for examinations performed, the results and the prognosis. Further, contradictory information with regard to the cause of their stroke and about their treatment was described *[53]*.*

The second is an example of Cognitive Participation; Legitimisation (CPLE). This demonstrates a mismatch in ideas and expectations between patients and health professionals and is categorised as ‘interacting with others’:

*For them recovery involved dimensions that were not included in the health care professionals’ concept….The goal for them was either to recapture their former social position or to adapt to another life situation *[54]*.*

The third is an example of Collective Action, Interactional Workability (CAIW). This describes inadequate patient services and would be categorised under ‘institutional admission’:

*Patients feel that therapy and supervised exercises in the ward facilitate regaining self-care, but they experience a lack of therapy and supervision, for example, when their therapist is ill or during weekends. In the patients’ view, this problem can be solved but patients find it difficult to judge *[55]*.*

Lastly, the fourth is an example of Reflexive Monitoring, Systemization (RMSY). This demonstrates routine self monitoring of progress, and would be categorised as ‘reflecting on management’:

*Mr Neville an 80 year old man set himself the target of walking unaided by the time he left hospital….He kept a diary of is progress which he made available to the research team *[56]*.*

Data on illness burden as opposed to treatment burden was excluded. The following is an excerpt from an included paper that demonstrates information about illness burden. This data was excluded:

*The following respondent focused on the fact that she was not able to perform activities as easily and quickly as she used to. Though she was able to do most of what she wanted to, the fact that she did it slower and with more effort than before was a constant source of frustration *[57]*.*

### Quality appraisal

Papers were generally of a reasonable quality: demonstrating that they had used information gained directly from patients themselves; displaying a clear explanation of methods used; and being transparent about generalisability. Aspects of quality less well demonstrated included: acknowledgment of the researchers influence on the analysis; and any note of conflicts of interest.

## Discussion

The vast and multifarious options available regarding methodological approaches for qualitative systematic review can make this process a challenging and creative task. Methods must therefore be explicitly described for transparency and reproducibility to be plausible and we have outlined the approach we adopted to maximise identification of eligible studies.

### Methodological challenges

Creating an appropriately sensitive and specific search strategy was a significant challenge, as we were essentially searching for a topic that has not previously been defined or indexed in a body of literature that itself is not adequately represented or indexed. From this point of view the scoping search was invaluable, as it provided key papers and key words that could be used to create the search strategy. We found that adding ‘qualitative methods’ as a concept made our search strategy considerably more specific while retaining sensitivity, as demonstrated by the return of all the key papers identified in the scoping search. Indeed, our final results showed that 94% of papers were identified by our predefined database search. This contrasts with the findings of Greenhalgh at al [25] who found only 30% of papers using this method. This could be explained by differences in the topic under review as well as in inclusion criteria with regards literature sources, or it could be an indication of differing sensitivities of the search strategies.

Another difficulty to be addressed was that we aimed to study a phenomenon that has not previously been conceptualised. Very few papers seek to understand treatment burden in chronic illness specifically, although information on this is made available through the investigation of the patient experience of disease management. For example, it is common for a paper to explore the patient experience of recovery after stroke, encompassing the illness trajectory itself and its affect on the patient’s lifeworld. Within the patient’s story there is often valuable information on treatment burden, although this may not have been the explicit aim of the study. Thus we are attempting to apply a conceptual framework to a set of studies that have used alternative theories and methods to analyse the patient experience.

A third issue was that data extraction was complex, as within each paper there was a significant amount of irrelevant qualitative data difficult to separate from that on treatment burden due to the difference in focus between the primary studies and the review. There was considerable data on illness rather than treatment burden, and on lifework burden such as managing the home or maintaining employment, carried out in parallel to but not as a direct consequence of the illness. This is in keeping with the milestone work published by Corbin and Strauss on the three lines of work experienced by those with chronic illness [58]. Such burdens all merit further exploration but were not the focus of our work. There was also frequent exploration of the patient’s views, ideas and expectations of services, although the material practices that resulted from this were often not explored or documented, leading to a limited insight into the patient’s world.

Fourth, the appropriateness and methodology of the quality appraisal of qualitative research is widely debated [27]. We decided to use a previously published method [17] which appealed to our desire for quality appraisal that can inform the overall analysis and discussion of the review, whilst avoiding the use of a formal checklist or scoring system that results in exclusion of studies [59]. The value of carrying out quality appraisal in this review is therefore debatable. It could be argued that it proved useful for enabling a better understanding of the included studies, and that appraisal would have highlighted any significant methodological flaws had any been present. There is evidence, however, that the appraisal of qualitative research is such a subjective process that reaching a strong agreement between researchers is unlikely [50,60]. This supports our decision not to exclude studies based on quality appraisal, but raises the issue of whether quality appraisal under these circumstances is a worthwhile process.

Finally, a particularly novel aspect of this review was our approach to data analysis. We analysed data using a coding framework underpinned by NPT, which has previously been shown to aid understanding of the organization and operationalisation of tasks (their implementation), how tasks are made into routine elements of everyday life (their embedding), and how practices are sustained and embedded into their social contexts (their integration) [43]. It has been successfully used to understand the ‘work’ involved in sickness careers [61] and to understand the treatment burden experienced by chronic heart failure patients [6]. We found this novel method of data analysis very useful and informative for identifying the components of treatment burden in chronic illness from the patient perspective. Our successful use of NPT in this context suggests that in addition to being useful for the analysis of primary studies, this theory lends itself suitably to the synthesis of qualitative studies [47]. Similar to other methods of framework analysis, this is particularly appropriate in the applied research arena, where *a priori* ideas and concepts exist yet researchers wish their findings to reflect themes that arise from within the data.

### Limitations/strengths

We limited our search to publications from the year 2000 and onwards. As our reviews are aimed at understanding the current patient experience of stroke, heart failure and diabetes management with the aim of informing current clinical practice and policy, it was deemed most pertinent to review the literature over the past decade. This reflects patient experiences of treatment burdens based on current health service practices rather than historical ones. Global management of these conditions has changed over time, for example, stroke management has changed greatly in recent years with the introduction of stroke units and community rehabilitation programs [62,63] and hence we believe this to be a reasonable approach but it could be viewed as a limitation. Also, we restricted our search to English language papers as we had no resources available for translation. There were no geographical restrictions set, but the language restriction will have imposed some geographical restrictions on our results. Important strengths are that we conducted an exhaustive search rather than a purposive approach, and the robust theoretical underpinning to our approach to data analysis. No formal assessments of sensitivity and specificity of our search strategy were carried out; specificity was estimated by assessment of the number of papers retrieved, and sensitivity by the return of all key papers identified in the scoping search. A more formal assessment would be essential to comprehensively validate the search strategy, and the absence of this could be considered a limitation.

All aspects of data extraction, quality appraisal and data analysis were carried out by two researchers, with a third party for disagreements. We chose to use this method to minimise bias on behalf of the researcher [64], and as a method of triangulation to enhance our analysis [15]. Our tight inclusion criteria allowed us to avoid collecting too broad a spectrum of methodologies, as high numbers of studies using extremely varied methods makes in depth analysis of the data and applicability of findings extremely challenging. Studies that were not in peer reviewed journals i.e. ‘grey literature’ were excluded to manage the scope of the review. This could be regarded as a limitation. Aspects of the screening process could be argued to be fairly subjective i.e. the inclusion of studies with roughly 30% or more relevant content. Bias was reduced by the use of two independent reviewers, both of whom had to answer ‘exclude’ based on the same criterion for a paper to be excluded. As a result, the number of studies included was considerable yet still feasible for the application of qualitative analysis. These exclusions and the exclusion of methodologies such as telephone and postal questionnaires could be regarded as limitations, as it is possible that some studies exploring treatment burden may have been missed, and it would be worthwhile for subsequent reviews to be carried out looking at these areas. These approaches helped us to maintain focus whilst producing a rich picture of stroke management.

The use of framework analysis in this systematic review was appropriate due to our *a priori* knowledge in this area. However, there is always a risk with framework analysis that data has been ‘shoe horned’ into the framework, with the possibility that some data may be missed. However, although this work was deductive to some extent, we were careful to augment the framework during analysis, being careful to ensure that our findings were derived directly from the data, and importantly, made a careful note of any data that fell outside of our framework. We failed to find any such data, which suggests that the use of NPT as the underpinning theory for our analysis proved to be appropriate in this case.

## Conclusion

We have described the methods used in one of three methodologically similar qualitative systematic reviews aimed at exploring treatment burden as experienced by patients with chronic disease. The exploration of a topic not previously conceptualised and the creation of our search strategy may be of interest to other researchers carrying out synthesis of qualitative studies. Importantly, the successful use of NPT to inform a coding frame for data analysis involving qualitative data that describes processes relating to self management highlights the potential of a new method for analysis of qualitative data within systematic reviews.

## Abbreviations

NPT: Normalization process theory.

## Competing interests

The authors declare that they have no competing interests.

## Authors’ contributions

KG, FSM, CRM, VMM, and GDB were all involved in the design of the reviews. PE created the search strategies with contribution from KG, FSM, VMM, and DE. KG, BJ, DM, SM, FSM, FS all screened papers, data extracted and analysed data. CRM analysed data. All authors read and approved the final manuscript.

## Author’s information

The International Minimally Disruptive Medicine Workgroup includes: Victor M Montori, Carl R May, Frances S Mair, Katie Gallacher, David Eton, Deborah Morrison, Bhautesh Jani, Sara Macdonald, Susan Browne, David Blane, Nilay Shah, Nathan Shipee, Patricia Erwin, Kathleen Yost.

## Pre-publication history

The pre-publication history for this paper can be accessed here:

http://www.biomedcentral.com/1471-2288/13/10/prepub

## Supplementary Material

Additional file 1 **Search strategy.** The full search strategy used in the stroke systematic review. Click here for file

Additional file 2 **Inclusion and exclusion criteria for papers.** Criteria used to include and exclude papers in the stroke systematic review. Click here for file

Additional file 3 **Inclusion and Exclusion criteria for data extraction.** Criteria used to include and exclude data within a paper in the stroke systematic review. Click here for file

Additional file 4 **Data extraction instrument.** The instrument used to extract data from papers included in the stroke systematic review. Click here for file

Additional file 5 **Quality appraisal instrument.** The instrument used to analyse the quality of papers included in the stroke systematic review, to inform discussion. Click here for file
