# Correction: Qualitative systematic reviews of treatment burden in stroke, heart failure and diabetes - Methodological challenges and solutions

**DOI:** 10.1186/1471-2288-13-160

**Published:** 2014-01-10

**Authors:** Katie Gallacher, Bhautesh Jani, Deborah Morrison, Sara Macdonald, David Blane, Patricia Erwin, Carl R May, Victor M Montori, David T Eton, Fiona Smith, G David Batty, Frances S Mair

**Affiliations:** 1 University of Glasgow, Scotland, UK; 2 Mayo Clinic, Rochester, MN, USA; 3 University of Southampton, England, UK; 4 University College London, England, UK

## Correction

After publication of the original article [1] it came to the publishers attention that the name of author G David Batty had been inadvertently transposed. The correct authors' list should read as indicated above. We apologize for any inconvenience this has caused.

## Pre-publication history

The pre-publication history for this paper can be accessed here:

http://www.biomedcentral.com/1471-2288/13/160/prepub
