# Supplementary material for: Qualitative systematic reviews of treatment burden in stroke, heart failure and diabetes - Methodological challenges and solutions
Source: BMC Med Res Methodol. 2013 Jan 28;13:10. doi: 10.1186/1471-2288-13-10 (PMC3568050; doi:10.1186/1471-2288-13-10)
Supplement: Additional file 1 — Search strategy. The full search strategy used in the stroke systematic review. [file 1471-2288-13-10-S1.doc]

Additional File 1 - Search Strategy

| **Ovid MEDLINE(R) In-Process & Other Non-Indexed Citations and Ovid MEDLINE(R) 1948 to Present # Searches Results Search Type**  1 *cerebrovascular disorders/ or exp *brain ischemia/ or exp "*intracranial embolism and thrombosis"/ or exp *intracranial hemorrhages/ or exp *stroke/ 131168 Advanced  2 *stroke/co, di, dh, dt, nu, pc, px, rh, th 17679 Advanced  3 (cerebrovascular disorders/co, di, dh, dt, nu, pc, px, rh, th or exp brain ischemia/co, di, dh, dt, nu, pc, px, rh, th or exp "intracranial embolism and thrombosis"/co, di, dh, dt, nu, pc, px, rh, th or exp intracranial hemorrhages/co, di, dh, dt, nu, pc, px, rh, th) and stroke*.mp. [mp=protocol supplementary concept, rare disease supplementary concept, title, original title, abstract, name of substance word, subject heading word, unique identifier] 24267 Advanced  4 1 or 2 or 3 133827 Advanced  5 limit 4 to (english language and humans and yr="2000 - 2011") 46196 Advanced  6 (burden* adj2 (treat* or therap*)).mp. [mp=protocol supplementary concept, rare disease supplementary concept, title, original title, abstract, name of substance word, subject heading word, unique identifier] 694 Advanced  7 activities of daily living/ or health behavior/ or health, knowledge, attitudes, practice/ or lifestyle/ or occupational therapy/ or palliat*.mp. or patient education as topic/ or exp rehabilitation/ or exp physical therapy modalities/ or self care/ or barthel*.mp. [mp=protocol supplementary concept, rare disease supplementary concept, title, original title, abstract, name of substance word, subject heading word, unique identifier] 395268 Advanced  8 Disabled Persons/ or Disability Evaluation/ or work capacity evaluation/ or functional disability.mp. 58737 Advanced  9 social support/ or exp family/px or exercise/ or "physical education and training"/ or physical endurance/ or fatigue/ or physical fitness/ or long term care/ 194957 Advanced  10 exp Gait/ or exp Gait Disorders, Neurologic/ or exp Accidental Falls/ 26855 Advanced  11 self administration/ or (treatment adj3 (regimen* or regime* or tasks or work)).mp. or unnecessary*.mp. or unmet.mp. [mp=protocol supplementary concept, rare disease supplementary concept, title, original title, abstract, name of substance word, subject heading word, unique identifier] 65409 Advanced  12 exp cognition disorders/ or independen*.mp. or dependenc*.mp. or socioeconomic factors/ or life change events/ [mp=protocol supplementary concept, rare disease supplementary concept, title, original title, abstract, name of substance word, subject heading word, unique identifier] 862844 Advanced  13 5 and 6 5 Advanced  14 5 and 7 4172 Advanced  15 5 and 8 1221 Advanced  16 5 and 9 694 Advanced  17 5 and 10 764 Advanced  18 5 and 11 244 Advanced  19 5 and 12 6432 Advanced  20 5 and (travel*.mp. or automobile driving/ or mobility limitation/ or transportation/) [mp=protocol supplementary concept, rare disease supplementary concept, title, original title, abstract, name of substance word, subject heading word, unique identifier] 168 Advanced  21 5 and (recover* or challeng* or goal*).mp. [mp=protocol supplementary concept, rare disease supplementary concept, title, original title, abstract, name of substance word, subject heading word, unique identifier] 5971 Advanced  22 5 and (interpersonal relations/ or social isolation/ or cost of illness/ or schedul*.mp.) [mp=protocol supplementary concept, rare disease supplementary concept, title, original title, abstract, name of substance word, subject heading word, unique identifier] 843 Advanced  23 or/13-22 15121 Advanced  24 (focus group* or ethnograph* or phenomenol* or observation* or (grounded adj theory) or (framework adj analysis) or (thematic adj analysis) or (constant adj comparison)).mp. [mp=protocol supplementary concept, rare disease supplementary concept, title, original title, abstract, name of substance word, subject heading word, unique identifier] 532412 Advanced  25 adaptation, psychological/ or adheren*.mp. or nonadheren*.mp. or patient compliance/ or noncomplian*.mp. or inconvenien*.mp. or negotiat*.mp. or (patient adj2 (care or experience or understand* or expectation* or perspective*)).mp. or patient satisfaction/ or personal autonomy/ or physician-patient relations/ or professional-patient relations/ or dissatis*.mp. or quality of life/ [mp=protocol supplementary concept, rare disease supplementary concept, title, original title, abstract, name of substance word, subject heading word, unique identifier] 503408 Advanced  26 self concept/ or self care/ or self-management/ or suffer*.mp. [mp=protocol supplementary concept, rare disease supplementary concept, title, original title, abstract, name of substance word, subject heading word, unique identifier] 226598 Advanced  27 attitude/ or attitude to health/ 99550 Advanced  28 (questionnaire* or survey* or qualitative* or interview*).mp. [mp=protocol supplementary concept, rare disease supplementary concept, title, original title, abstract, name of substance word, subject heading word, unique identifier] 800092 Advanced  29 23 and (25 or 26 or 27) and (24 or 28) 730 Advanced  30 23 and (exp leisure activities/ or health status/ or well-being.mp.) and (24 or 28) [mp=protocol supplementary concept, rare disease supplementary concept, title, original title, abstract, name of substance word, subject heading word, unique identifier] 203 Advanced  31 29 or 30 821 Advanced  32 Health Services Accessibility/ 39127 Advanced  33 23 and (32 or continuity of patient care/ or disrupt*.mp.) and (24 or 28) [mp=protocol supplementary concept, rare disease supplementary concept, title, original title, abstract, name of substance word, subject heading word, unique identifier] 89 Advanced  34 5 and (communication* or literac*).mp. and (24 or 28) [mp=protocol supplementary concept, rare disease supplementary concept, title, original title, abstract, name of substance word, subject heading word, unique identifier] 161 Advanced  35 31 or 33 or 34 958 Advanced  36 5 and sickness impact profile/ 101 Advanced  37 5 and needs assessment/ 117 Advanced  38 35 or 36 or 37 1088  1084 after duplicates removed.  **EMBASE 1988 to 2011 Week 09 # Searches Results Search Type**  1 *cerebrovascular disorders/ or exp *brain ischemia/ or exp "*intracranial embolism and thrombosis"/ or exp *intracranial hemorrhages/ or exp *stroke/ 98693 Advanced  2 *stroke/co, di, dm, dt, rh, th 18765 Advanced  3 cerebrovascular disorders/co, di, dm, dt, rh, th or brain ischemia/co, di, dm, dt, rh, th or exp "intracranial embolism and thrombosis"/co, di, dm, dt, dm, rh, th or exp intracranial hemorrhages/co, di, dm, dt, rh, th 140085 Advanced  4 1 or 2 or 3 209703 Advanced  5 limit 4 to (english language and humans and yr="2000 - 2011") 96878 Advanced  6 ((burden* adj2 (treat* or therap*)) or hassle* or medication adherence or medication compliance or medication concordance or physiotherapy or ocupational therapy).mp. or rehabilitation/ [mp=title, abstract, subject headings, heading word, drug trade name, original title, device manufacturer, drug manufacturer] 54538 Advanced  7 activities of daily living/ or health behavior/ or health, knowledge, attitudes, practice/ or lifestyle/ or occupational therapy/ or palliat*.mp. or patient education as topic/ or exp rehabilitation/ or exp physical therapy modalities/ or self care/ or barthel*.mp. [mp=title, abstract, subject headings, heading word, drug trade name, original title, device manufacturer, drug manufacturer] 431790 Advanced  8 Disabled Persons/ or Disability Evaluation/ or work capacity evaluation/ or functional disability.mp. 56987 Advanced  9 social support/ or exp family/px or exercise/ or "physical education and training"/ or physical endurance/ or fatigue/ or physical fitness/ or long term care/ 285571 Advanced  10 exp Gait/ or exp Gait Disorders, Neurologic/ or exp Accidental Falls/ 1497271 Advanced  11 self administration/ or (treatment adj3 (regimen* or regime* or tasks or work)).mp. or unnecessary*.mp. or unmet.mp. [mp=title, abstract, subject headings, heading word, drug trade name, original title, device manufacturer, drug manufacturer] 65351 Advanced  12 exp cognition disorders/ or independen*.mp. or dependenc*.mp. or socioeconomic factors/ or life change events/ [mp=title, abstract, subject headings, heading word, drug trade name, original title, device manufacturer, drug manufacturer] 880442 Advanced  13 5 and 6 1295 Advanced  14 5 and 7 5870 Advanced  15 5 and 8 1301 Advanced  16 5 and 9 3950 Advanced  17 5 and 10 56698 Advanced  18 5 and 11 721 Advanced  19 5 and 12 8891 Advanced  20 5 and (travel*.mp. or automobile driving/ or mobility limitation/ or transportation/) [mp=title, abstract, subject headings, heading word, drug trade name, original title, device manufacturer, drug manufacturer] 415 Advanced  21 5 and (recover* or challeng* or goal*).mp. [mp=title, abstract, subject headings, heading word, drug trade name, original title, device manufacturer, drug manufacturer] 8316 Advanced  22 5 and (interpersonal relations/ or social isolation/ or cost of illness/ or schedul*.mp.) [mp=title, abstract, subject headings, heading word, drug trade name, original title, device manufacturer, drug manufacturer] 1033 Advanced  23 or/13-22 63985 Advanced  24 (focus group* or ethnograph* or phenomenol* or observation* or (grounded adj theory) or (framework adj analysis) or (thematic adj analysis) or (constant adj comparison)).mp. [mp=title, abstract, subject headings, heading word, drug trade name, original title, device manufacturer, drug manufacturer] 460592 Advanced  25 adaptation, psychological/ or adheren*.mp. or nonadheren*.mp. or patient compliance/ or noncomplian*.mp. or inconvenien*.mp. or negotiat*.mp. or (patient adj2 (care or experience or understand* or expectation* or perspective*)).mp. or patient satisfaction/ or personal autonomy/ or physician-patient relations/ or professional-patient relations/ or dissatis*.mp. or quality of life/ [mp=title, abstract, subject headings, heading word, drug trade name, original title, device manufacturer, drug manufacturer] 626175 Advanced  26 self concept/ or self care/ or self-management/ or suffer*.mp. [mp=title, abstract, subject headings, heading word, drug trade name, original title, device manufacturer, drug manufacturer] 233056 Advanced  27 attitude/ or attitude to health/ 85807 Advanced  28 (questionnaire* or survey* or qualitative* or interview*).mp. [mp=title, abstract, subject headings, heading word, drug trade name, original title, device manufacturer, drug manufacturer] 1111075 Advanced  29 23 and (25 or 26 or 27) and (24 or 28) 1315 Advanced  30 23 and (exp leisure activities/ or health status/ or well-being.mp.) and (24 or 28) [mp=title, abstract, subject headings, heading word, drug trade name, original title, device manufacturer, drug manufacturer] 221 Advanced  31 29 or 30 1413 Advanced  32 Health Services Accessibility/ 88876 Advanced  33 23 and (32 or continuity of patient care/ or disrupt*.mp.) and (24 or 28) [mp=title, abstract, subject headings, heading word, drug trade name, original title, device manufacturer, drug manufacturer] 370 Advanced  34 5 and (communication* or literac*).mp. and (24 or 28) [mp=title, abstract, subject headings, heading word, drug trade name, original title, device manufacturer, drug manufacturer] 170 Advanced  35 31 or 33 or 34 1587 Advanced  36 5 and sickness impact profile/ 20 Advanced  37 5 and needs assessment/ 29 Advanced  38 35 or 36 or 37 1609 Advanced  39 limit 38 to (human and yr="2000 - 2011") 1609 Advanced  40 39 not case report/ 1554 Advanced  41 ((experience or recovery or service or patient*) adj3 stroke*).mp. [mp=title, abstract, subject headings, heading word, drug trade name, original title, device manufacturer, drug manufacturer] 40540 Advanced  42 (exercise or lifestyle or patient education* or social support or social isolation or self care or burden or community support or cost of illness or drug costs or imipact or inconvenience or negotiat* or patient experience or patient perspective or patient preference or patient satisfaction or dissatis*).mp. or professional-patient relations/ or physician patient relations.mp. or quality of life.mp. or recovery*.mp. or suffer*.mp. [mp=title, abstract, subject headings, heading word, drug trade name, original title, device manufacturer, drug manufacturer] 1092852 Advanced  43 5 and (41 or 42) 21385 Advanced  44 43 and 24 968 Advanced  45 39 or 44 2226 Advanced  46 (*cerebrovascular disorders/ or exp *brain ischemia/ or exp *"*intracranial embolism and thrombosis"/ or exp *intracranial hemorrhages/ or exp *stroke/) and 45 1575  **PsycINFO 1987 to March Week 1 2011 # Searches Results Search Type**  1 *cerebrovascular disorders/ or exp *brain ischemia/ or exp "*intracranial embolism and thrombosis"/ or exp *intracranial hemorrhages/ or exp *stroke/ 8554 Advanced  2 [*stroke/co, di, dm, dt, rh, th] 0 Advanced  3 [cerebrovascular disorders/co, di, dm, dt, rh, th or brain ischemia/co, di, dm, dt, rh, th or exp "intracranial embolism and thrombosis"/co, di, dm, dt, dm, rh, th or exp intracranial hemorrhages/co, di, dm, dt, rh, th] 0 Advanced  4 1 or 2 or 3 8554 Advanced  5 limit 4 to (english language and humans and yr="2000 - 2011") [Limit not valid in PsycINFO; records were retained] 6124 Advanced  6 ((burden* adj2 (treat* or therap*)) or hassle* or medication adherence or medication compliance or medication concordance or physiotherapy or ocupational therapy).mp. or rehabilitation/ [mp=title, abstract, heading word, table of contents, key concepts] 12789 Advanced  7 activities of daily living/ or health behavior/ or health, knowledge, attitudes, practice/ or lifestyle/ or occupational therapy/ or palliat*.mp. or patient education as topic/ or exp rehabilitation/ or exp physical therapy modalities/ or self care/ or barthel*.mp. [mp=title, abstract, heading word, table of contents, key concepts] 64665 Advanced  8 Disabled Persons/ or Disability Evaluation/ or work capacity evaluation/ or functional disability.mp. 1280 Advanced  9 social support/ or exp family/px or exercise/ or "physical education and training"/ or physical endurance/ or fatigue/ or physical fitness/ or long term care/ 36627 Advanced  10 exp Gait/ or exp Gait Disorders, Neurologic/ or exp Accidental Falls/ 0 Advanced  11 self administration/ or (treatment adj3 (regimen* or regime* or tasks or work)).mp. or unnecessary*.mp. or unmet.mp. [mp=title, abstract, heading word, table of contents, key concepts] 8552 Advanced  12 exp cognition disorders/ or independen*.mp. or dependenc*.mp. or socioeconomic factors/ or life change events/ [mp=title, abstract, heading word, table of contents, key concepts] 123081 Advanced  13 5 and 6 613 Advanced  14 5 and 7 1017 Advanced  15 5 and 8 30 Advanced  16 5 and 9 137 Advanced  17 5 and 10 0 Advanced  18 5 and 11 35 Advanced  19 5 and 12 850 Advanced  20 5 and (travel*.mp. or automobile driving/ or mobility limitation/ or transportation/) [mp=title, abstract, heading word, table of contents, key concepts] 15 Advanced  21 5 and (recover* or challeng* or goal*).mp. [mp=title, abstract, heading word, table of contents, key concepts] 1256 Advanced  22 5 and (interpersonal relations/ or social isolation/ or cost of illness/ or schedul*.mp.) [mp=title, abstract, heading word, table of contents, key concepts] 36 Advanced  23 or/13-22 2565 Advanced  24 (focus group* or ethnograph* or phenomenol* or observation* or (grounded adj theory) or (framework adj analysis) or (thematic adj analysis) or (constant adj comparison)).mp. [mp=title, abstract, heading word, table of contents, key concepts] 106510 Advanced  25 adaptation, psychological/ or adheren*.mp. or nonadheren*.mp. or patient compliance/ or noncomplian*.mp. or inconvenien*.mp. or negotiat*.mp. or (patient adj2 (care or experience or understand* or expectation* or perspective*)).mp. or patient satisfaction/ or personal autonomy/ or physician-patient relations/ or professional-patient relations/ or dissatis*.mp. or quality of life/ [mp=title, abstract, heading word, table of contents, key concepts] 64081 Advanced  26 self concept/ or self care/ or self-management/ or suffer*.mp. [mp=title, abstract, heading word, table of contents, key concepts] 66063 Advanced  27 attitude/ or attitude to health/ 0 Advanced  28 (questionnaire* or survey* or qualitative* or interview*).mp. [mp=title, abstract, heading word, table of contents, key concepts] 372484 Advanced  29 23 and (25 or 26 or 27) and (24 or 28) 110 Advanced  30 23 and (exp leisure activities/ or health status/ or well-being.mp.) and (24 or 28) [mp=title, abstract, heading word, table of contents, key concepts] 18 Advanced  31 29 or 30 121 Advanced  32 Health Services Accessibility/ 0 Advanced  33 23 and (32 or continuity of patient care/ or disrupt*.mp.) and (24 or 28) [mp=title, abstract, heading word, table of contents, key concepts] 11 Advanced  34 5 and (communication* or literac*).mp. and (24 or 28) [mp=title, abstract, heading word, table of contents, key concepts] 67 Advanced  35 31 or 33 or 34 184 Advanced  36 5 and sickness impact profile/ 0 Advanced  37 5 and needs assessment/ 3 Advanced  38 35 or 36 or 37 187 Advanced  39 limit 38 to (human and yr="2000 - 2011") 185 Advanced  40 39 not case report/ 185 Advanced  41 ((experience or recovery or service or patient*) adj3 stroke*).mp. [mp=title, abstract, heading word, table of contents, key concepts] 4084 Advanced  42 (exercise or lifestyle or patient education* or social support or social isolation or self care or burden or community support or cost of illness or drug costs or imipact or inconvenience or negotiat* or patient experience or patient perspective or patient preference or patient satisfaction or dissatis*).mp. or professional-patient relations/ or physician patient relations.mp. or quality of life.mp. or recovery*.mp. or suffer*.mp. [mp=title, abstract, heading word, table of contents, key concepts] 194242 Advanced  43 5 and (41 or 42) 3299 Advanced  44 43 and 24 199 Advanced  45 39 or 44 345 Advanced  46 limit 45 to all journals 321  **CINAHL**  S22 or S27 Limiters - Published Date from: 20000101-20111231; English Language; Human 325  Search modes - Boolean/Phrase  View Results (325) View Details Edit Interface - EBSCOhost  Search Screen - Advanced Search  Database - CINAHL  S28 S22 or S27 Search modes - Boolean/Phrase  View Results (433) View Details Edit Interface - EBSCOhost  Search Screen - Advanced Search  Database - CINAHL  S27 S1 and S26 and S11 Search modes - Boolean/Phrase  View Results (394) View Details Edit Interface - EBSCOhost  Search Screen - Advanced Search  Database - CINAHL  S26 S23 or S24 or S25 Search modes - Boolean/Phrase  View Results (675047) View Details Edit Interface - EBSCOhost  Search Screen - Advanced Search  Database - CINAHL  S25 disappoint* or dissatisf* or emotional or eating or frustrat* Search modes - Boolean/Phrase  View Results (39771) View Details Edit Interface - EBSCOhost  Search Screen - Advanced Search  Database - CINAHL  S24 (MH "Quality of Life+") OR (MH "Quality of Working Life") OR (MH "Quality of Life (Iowa NOC)") OR (MH "Attitude to Life") Search modes - Boolean/Phrase  View Results (34692) View Details Edit Interface - EBSCOhost  Search Screen - Advanced Search  Database - CINAHL  S23 function* or negotiat* or patient* Search modes - Boolean/Phrase  View Results (638185) View Details Edit Interface - EBSCOhost  Search Screen - Advanced Search  Database - CINAHL  S22 S13 or S21 Search modes - Boolean/Phrase  View Results (199) View Details Edit Interface - EBSCOhost  Search Screen - Advanced Search  Database - CINAHL  S21 S1 and S11 and S20 Search modes - Boolean/Phrase  View Results (135) View Details Edit Interface - EBSCOhost  Search Screen - Advanced Search  Database - CINAHL  S20 S14 or S15 or S16 or S18 or S19 Search modes - Boolean/Phrase  View Results (107052) View Details Edit Interface - EBSCOhost  Search Screen - Advanced Search  Database - CINAHL  S19 (MH "Recovery") Search modes - Boolean/Phrase  View Results (8333) View Details Edit Interface - EBSCOhost  Search Screen - Advanced Search  Database - CINAHL  S18 (MH "Patient Satisfaction") Search modes - Boolean/Phrase  View Results (18386) View Details Edit Interface - EBSCOhost  Search Screen - Advanced Search  Database - CINAHL  S17 "patient experience" or "patient satisfaction" or "patient dissatisf* Search modes - Boolean/Phrase  View Results (0) View Details Edit Interface - EBSCOhost  Search Screen - Advanced Search  Database - CINAHL  S16 (MH "Patient Education+") OR (MH "Patient Discharge Education") OR (MH "Patient Education (Iowa NIC) (Non-Cinahl)+") Limiters - Published Date from: 20000101-20111231; English Language  Search modes - Boolean/Phrase  View Results (24855) View Details Edit Interface - EBSCOhost  Search Screen - Advanced Search  Database - CINAHL  S15 (MH "Patient Care Plans+") OR (MH "Discharge Planning+") Limiters - Published Date from: 20000101-20111231; English Language  Search modes - Boolean/Phrase  View Results (4000) View Details Edit Interface - EBSCOhost  Search Screen - Advanced Search  Database - CINAHL  S14 (MH "Life Style+") Limiters - Published Date from: 20000101-20111231; English Language  Search modes - Boolean/Phrase  View Results (56964) View Details Edit Interface - EBSCOhost  Search Screen - Advanced Search  Database - CINAHL  S13 S9 and S11 Limiters - Published Date from: 20000101-20111231; English Language  Search modes - Boolean/Phrase  View Results (108) View Details Edit Interface - EBSCOhost  Search Screen - Advanced Search  Database - CINAHL  S12 S9 and S11 Search modes - Boolean/Phrase  View Results (128) View Details Edit Interface - EBSCOhost  Search Screen - Advanced Search  Database - CINAHL  S11 (MH "Qualitative Studies+") OR (MH "Clinical Nursing Research") OR (MH "Clinical Research+") Search modes - Boolean/Phrase  View Results (56296) View Details Edit Interface - EBSCOhost  Search Screen - Advanced Search  Database - CINAHL  S10 ch Search modes - Boolean/Phrase  View Results (0) View Details Edit Interface - EBSCOhost  Search Screen - Advanced Search  Database - CINAHL  S9 S1 and S8 Search modes - Boolean/Phrase  View Results (1654) View Details Edit Interface - EBSCOhost  Search Screen - Advanced Search  Database - CINAHL  S8 S2 or S3 or S4 or S5 or S6 or S7 Search modes - Boolean/Phrase  View Results (99528) View Details Edit Interface - EBSCOhost  Search Screen - Advanced Search  Database - CINAHL  S7 (MH "Social Support (Iowa NOC)") OR (MH "Norbeck Social Support Questionnaire") OR (MH "Social Support Index") OR (MH "Support, Psychosocial+") OR (MH "Coping Support (Saba CCC)") Search modes - Boolean/Phrase  View Results (28536) View Details Edit Interface - EBSCOhost  Search Screen - Advanced Search  Database - CINAHL  S6 lifestyle Search modes - Boolean/Phrase  View Results (10643) View Details Edit Interface - EBSCOhost  Search Screen - Advanced Search  Database - CINAHL  S5 (MH "Health Knowledge") Search modes - Boolean/Phrase  View Results (10537) View Details Edit Interface - EBSCOhost  Search Screen - Advanced Search  Database - CINAHL  S4 (MH "Health Behavior+") OR (MH "Health Behavior Component (Saba CCC)+") OR (MH "Health Seeking Behavior Alteration (Saba CCC)") OR (MH "Domain IV: Health-Related Behaviors Domain (Omaha)+") OR (MH "Health Behavior (Iowa NOC) (Non-Cinahl)+") OR (MH "Health Knowledge and Behavior (Iowa NOC) (Non-Cinahl)+") OR (MH "Health Promoting Behavior (Iowa NOC)") Search modes - Boolean/Phrase  View Results (34570) View Details Edit Interface - EBSCOhost  Search Screen - Advanced Search  Database - CINAHL  S3 "burden of treatment" or "burden of therapy" or hassle* or inconvenien* or "treatment burden" Search modes - Boolean/Phrase  View Results (1023) View Details Edit Interface - EBSCOhost  Search Screen - Advanced Search  Database - CINAHL  S2 (MH "Altered Activities of Daily Living (NANDA) (Non-Cinahl)+") OR (MH "Self Care: Activities of Daily Living (Iowa NOC)") OR (MH "Self-Care: Instrumental Activities of Daily Living (Iowa NOC)") OR (MH "Activities of Daily Living+") OR (MH "Activities of Daily Living (Saba CCC)") OR (MH "Activities of Daily Living Alteration (Saba CCC)") OR (MH "Instrumental Activities of Daily Living (Saba CCC)") OR (MH "Instrumental Activities of Daily Living Alteration (Saba CCC)") Search modes - Boolean/Phrase  View Results (21685) View Details Edit Interface - EBSCOhost  Search Screen - Advanced Search  Database - CINAHL  S1 (MM "Stroke") OR (MM "Stroke Patients") Search modes - Boolean/Phrase  View Results (17257) View Details Edit  CINAHL = 514   Search ID#  Search Terms  Search Options  Actions      S15   S9 or S14   Search modes - Boolean/Phrase     View Results  (514)     S14   S1 and S11   Limiters - Published Date from: 20000101-20111231; English Language; Peer Reviewed; Research Article     View Results  (125)     S13   S1 and S11   Limiters - Published Date from: 20000101-20111231; English Language; Peer Reviewed; Research Article     S12   S1 and S11   Search modes - Boolean/Phrase     View Results  (184)     S11   (MH "patient centered care") or (MH "community health services") or (MH "health resource utilization") or (MH "communicty reintegration") or (MH "patient attitudes") or (MH "recovery") or (MH "patient satisfaction")   Search modes - Boolean/Phrase     View Results  (60433)     S10   S1 and S6   Search modes - Boolean/Phrase     View Results  (647)     S9   S6 and S7   Limiters - Published Date from: 20000101-20111231; English Language; Peer Reviewed; Exclude MEDLINE records     View Results  (405)     S8   S6 and S7   Search modes - Boolean/Phrase     View Results  (3118)     S7   S1 or S2   Search modes - Boolean/Phrase     View Results  (17579)     S6   S3 or S4 or S5   Search modes - Boolean/Phrase     View Results  (238050)     S5   (MH "Interviews+")   Search modes - Boolean/Phrase     View Results  (90635)     S4   (MH "Scales") OR (MH "Questionnaires+")   Search modes - Boolean/Phrase     S3   (MH "Qualitative Studies+")   Search modes - Boolean/Phrase     View Results  (49055)     S2   (MM "Stroke")   Search modes - Boolean/Phrase     View Results  (16899)     S1   (MH "Stroke Patients")   Search modes - Boolean/Phrase  1692  **SCOPUS** TITLE-ABS-KEY-AUTH(stroke AND burden* AND (rehab* OR poststroke)) AND PUBYEAR AFT 1999  TITLE-ABS-KEY((stroke* OR poststroke OR "cerebrovascular accident*")) AND ((rehabilitat* OR satisfact* OR dissatisfact* OR recover* OR surviv* OR work OR occupation* OR "return to work" OR community OR support OR recover* OR independen* OR dependen* OR driving OR coping OR frustrat* OR attitude* OR communicat* OR activities* OR adapt* OR burden* OR self OR impact OR inconven* OR mood* OR emotion* OR sexual* OR eating OR dysphag* OR dysfunction* OR disappoint* OR expectation* OR intimacy OR incontinen* OR depress* OR fatigue* OR preference OR quality OR suffer* OR unmet) AND (qualitative* OR survey* OR interview* OR questionnaire* OR perspective* OR scale* OR narrative* OR focus OR observational OR framework OR thematic OR "grounded theory" OR population* OR cohort* OR follow*)) AND TITLE(aphasi* OR "post-stroke" OR poststroke OR (stroke AND (surviv* OR recover* OR rehabil* OR needs OR goal* OR patient* OR surviv*))) AND PUBYEAR AFT 1999 AND LANGUAGE(english) AND NOT (PMID(1* OR 2* OR 3* OR 4* OR 5* OR 6* OR 7* OR 8* OR 9*)) AND NOT TITLE-ABS-KEY("case report*" OR mice OR rats) AND (EXCLUDE(DOCTYPE, "ip")) AND (EXCLUDE(SUBJAREA, "ENGI"))  1619 |
| --- |
