# Supplementary material for: Qualitative systematic reviews of treatment burden in stroke, heart failure and diabetes - Methodological challenges and solutions
Source: BMC Med Res Methodol. 2013 Jan 28;13:10. doi: 10.1186/1471-2288-13-10 (PMC3568050; doi:10.1186/1471-2288-13-10)
Supplement: Additional file 3 — Inclusion and Exclusion criteria for data extraction. Criteria used to include and exclude data within a paper in the stroke systematic review. [file 1471-2288-13-10-S3.doc]

Additional File 3 – Inclusion and Exclusion criteria for data extraction.

| **Inclusion criteria** | **Examples** |
| --- | --- |
| Treatment burdens arising due to the demands of illness management | ‘In caring for self, stroke survivors had reached the point of performing certain tasks and activities without help from others’. Eaves |
| Treatment burdens arising due to service insufficiencies | ‘Mr X (who, in addition to the stroke, suffered heart problems and had one leg amputated below the knee) spent over a thousand nights sleeping in a chair downstairs waiting for the housing department’. Hart |
| **Exclusion criteria** | **Examples** |
| Illness burdens such as symptoms or physical consequences of illness, changes to relationships due to illness, psychological consequences of illness. | ‘Some of them had become physically handicapped and some had become more forgetful, while others suffered from fatigue’. Bendz |
|  | ‘A number of people said that they tended  not to tell anyone about their stroke unless they were family members or close friends because of perceived stigmatizing responses, which added to their psychological and physical isolation’. Hare |
| Lifework burdens such as those caused by circumstances and commitments out with the illness. | ‘Mr x lived in a fairly isolated area with minimal public transport and his mobility was very limited until he was able to drive again. He thus experienced exclusion from his local community.’ Alaszewski |
| General comments on services and service deficits where no resultant treatment burden is discussed or no specific negative encounter has been endured. | ‘One participant in the home setting discussed the importance of physiotherapy to her overall rehabilitation’ Hale |
|  | ‘Those interviewed expressed a great deal of sympathy and understanding for the health professionals that had cared and rehabilitated them. All of the informants talked about the current stress in society and how it affected them and health professionals. Although the informants were critical of the rehabilitation, they showed understanding of the stressful situation in hospital work’. Roding |
| Comments on patient perception for the purpose of the research project rather than for the purpose of stroke management. | In contrast to the very exact and detailed descriptions of falling ill most of the patients described very briefly and superficially their time in care’. Olofsson |
| Patient ideas and expectations. | ‘the informant during his rehabilitation had a need for a reflected understanding of what rehabilitation meant to him when all his experiences were included’. Hjelmblink |
