# Supplementary material for: Qualitative systematic reviews of treatment burden in stroke, heart failure and diabetes - Methodological challenges and solutions
Source: BMC Med Res Methodol. 2013 Jan 28;13:10. doi: 10.1186/1471-2288-13-10 (PMC3568050; doi:10.1186/1471-2288-13-10)
Supplement: Additional file 4 — Data extraction instrument. The instrument used to extract data from papers included in the stroke systematic review. [file 1471-2288-13-10-S4.doc]

Additional File 4 – Data Extraction Instrument (with example of qualitative data extraction)

| **ARTICLE DETAILS** | | | | |  |
| --- | --- | --- | --- | --- | --- |
| Study Title. |  | | | |  |
| Authors. |  | | | |  |
| Journal, Vol, Issue, Page nos. |  | | | |  |
| Year. |  | | | |  |
| DOI / artice ID |  | | | |  |
| Setting | community | | |  |  |
|  | hospital inpatient (general or unspecified ward) | | |  |  |
|  | stroke unit | | |  |  |
|  | rehab ward | | |  |  |
|  | care home | | |  |  |
|  | outpatient rehabilitation centre | | |  |  |
|  | outpatient clinic | | |  |  |
|  | other | | |  |  |
|  | unclear | | |  |  |
| **STUDY DETAILS** | **Provided** | **Not provided** | **Unclear** | |  |
| What is the research question or research objective/ s? (e.g. to find out how stroke patients manage their illness?) |  |  |  | |  |
| How are participants sampled? (e.g. theoretical, purposive, random. |  |  |  | |  |
| How is data collected? (e.g. interviews, questionnaires.) |  |  |  | |  |
| How is data analysed? (e.g. grounded theory, thematic analysis.) |  |  |  | |  |
| What is the overall conclusion or recommendations of the study? |  |  |  | |  |
| What (if any) study limitations are declared? |  |  |  | |  |
| How is the study funded? Are any conflicts of interest declared? |  |  |  | |  |
| **PARTICIPANT DETAILS** | | | | |  |
| Inclusion criteria |  | | | |  |
| Exclusion criteria |  | | | |  |
| Number of participants |  | | | |  |
| Min age of participants |  | | | |  |
| Max age if participants |  | | | |  |
| Mean age of participants |  | | | |  |
| Number of males |  | | | |  |
| Number of females |  | | | |  |
| Employment | employed | | |  |  |
|  | unemployed | | |  |  |
|  | retired | | |  |  |
|  | other | | |  |  |
|  | unclear | | |  |  |
| Marital status | married / civil partnership | | |  |  |
|  | living with partner | | |  |  |
|  | divorced | | |  |  |
|  | separated | | |  |  |
|  | widowed | | |  |  |
|  | single | | |  |  |
|  | other | | |  |  |
|  | unclear | | |  |  |
| Socioeconomic status |  | | | |  |
| Type of housing | owned | | |  |  |
|  | council | | |  |  |
|  | care home | | |  |  |
|  | private rent | | |  |  |
|  | lives with home owner | | |  |  |
|  | other | | |  |  |
|  | unclear | | |  |  |
|  | warden supervised | | |  |  |
| Ethnicity | white british | | |  |  |
|  | white european | | |  |  |
|  | white american | | |  |  |
|  | white other | | |  |  |
|  | african american | | |  |  |
|  | native american indian | | |  |  |
|  | black caribbean | | |  |  |
|  | black african | | |  |  |
|  | chinese | | |  |  |
|  | asian | | |  |  |
|  | mixed | | |  |  |
|  | other | | |  |  |
|  | unclear | | |  |  |
| Comorbidities | yes | | |  |  |
|  | no | | |  |  |
|  | unknown | | |  |  |
|  | unclear | | |  |  |
| Min number of co-morbidities |  | | | |  |
| Max number of co-morbidities |  | | | |  |
| Types of co-morbidities | hypertension | | |  |  |
|  | CHD | | |  |  |
|  | diabetes | | |  |  |
|  | asthma | | |  |  |
|  | COPD | | |  |  |
|  | chronic renal failure | | |  |  |
|  | PVD | | |  |  |
|  | TIA | | |  |  |
|  | previous stroke | | |  |  |
|  | other | | |  |  |
|  | unclear | | |  |  |
| Min number of medications |  | | | |  |
| Max number of medications |  | | | |  |
| Mean number of medications |  | | | |  |
| Mobility | idependently mobile | | |  |  |
|  | walks with stick | | |  |  |
|  | walks with frame | | |  |  |
|  | wheelchair | | |  |  |
|  | other | | |  |  |
|  | unclear | | |  |  |
| Min time since stroke |  | | | |  |
| Max time simce stroke |  | | | |  |
| Mean time since stroke |  | | | |  |
| Min time in hopsital |  | | | |  |
| Max time in hopsital |  | | | |  |
| Mean time in hospital |  | | | |  |
| Pathophysiology of stroke | infarct | | |  |  |
|  | intracerebral haemorrhage | | |  |  |
|  | subarachnoid haemorrhage | | |  |  |
|  | other | | |  |  |
|  | unclear | | |  |  |
| Anatomical type of stroke | total anterior | | |  |  |
|  | partial anterior | | |  |  |
|  | posterior | | |  |  |
|  | lacunar | | |  |  |
|  | brainstem | | |  |  |
|  | left | | |  |  |
|  | right | | |  |  |
|  | other | | |  |  |
|  | unclear | | |  |  |
| Physical disability | right hemiplegia | | |  |  |
|  | left hemiplegia | | |  |  |
|  | visual field defect | | |  |  |
|  | sensory deficit | | |  |  |
|  | neglect | | |  |  |
|  | dysphagia | | |  |  |
|  | other | | |  |  |
|  | unclear | | |  |  |
| Speech problems | Dysphasia | | |  |  |
|  | Dysarthria | | |  |  |
|  | Other | | |  |  |
|  | Unclear | | |  |  |
|  | aphasia | | |  |  |
| Cognition | memory problems | | |  |  |
|  | disinhibition | | |  |  |
|  | confusion | | |  |  |
|  | other | | |  |  |
|  | unclear | | |  |  |
| Psychological consequences | low mood | | |  |  |
|  | fatigue | | |  |  |
|  | poor motivation | | |  |  |
|  | poor concentration | | |  |  |
|  | anxiety | | |  |  |
|  | other | | |  |  |
|  | unclear | | |  |  |
| **QUOTE** | **NPT code** | | | **TYPES OF TREATMENT WORK** | |
|  |  | | |  | |
|  |  | | |  | |
