# Supplementary material for: Qualitative systematic reviews of treatment burden in stroke, heart failure and diabetes - Methodological challenges and solutions
Source: BMC Med Res Methodol. 2013 Jan 28;13:10. doi: 10.1186/1471-2288-13-10 (PMC3568050; doi:10.1186/1471-2288-13-10)
Supplement: Additional file 5 — Quality appraisal instrument. The instrument used to analyse the quality of papers included in the stroke systematic review, to inform discussion. [file 1471-2288-13-10-S5.doc]

Additional file 5 – Quality Appraisal Instrument

| **Appraisal Question (apply each question to the whole study to reach an overall conclusion i.e. aims, sampling, data collection, data analysis, interpretations)** |
| --- |
| *Does the research, as reported, illuminate the subjective meaning, actions, and context of those being researched?*  i.e. is it ensured through design and analysis that emphasis is given to the interpretations of those being researched rather than the researcher’s or professional’s viewpoint? |
| *Are subjective perceptions and experiences treated as knowledge in their own right?*  i.e. does the study treat the data collected directly from the participants, representing their viewpoint, as the basic data for analysis? |
| *Is there evidence of the adaption and responsiveness of the research design to the circumstances and issues of real-life social settings met during the course of the study?*  i.e. is the process of sampling, data collection, data analysis and interpretation iterative? Is there evidence of adaption and redesign as the study has progressed? |
| *Does the sample produce the type of knowledge necessary to understand the structures and processes within which the individuals or situations are located?*  i.e. is sampling appropriate for the aims, objectives, methods and conclusions reached? |
| *Is the description provided detailed enough to allow the researcher or reader to interpret the meaning and context of what is being researched?*  i.e. is a rich picture produced, providing the context of an experience and the intentions and meanings that feed into it, rather than simply a set of facts? |
| *Are any different sources of knowledge about the same issue compared and contrasted and how is this done?*  i.e. are different methods used to answer the research question and are these examined not only for similarities but for providing different facets of the reality being investigated? |
| *Has the researcher rendered transparent the processes by which data have been collected, analyzed, and presented?*  i.e. is the whole process clear to the reader? |
| *Has the researcher made clear their own possible influence on the data?*  i.e. has the researcher stated their own background / experience and ontological / epistemological stance? |
| *Is it clear how the research moves from a description of the data, through quotation or examples, to an analysis and interpretation of the meaning and significance of it?*  i.e. what did the researcher do to reach their conclusions and does this make sense from the original data? |
| *Are claims being made for the generalizability of the findings to either other bodies of knowledge or to other populations or groups and if so what are these claims?*  i.e. is it made clear which settings the findings can be applied to and does this seem to fit? |
| *Is there any other aspect of the study that may affect the quality e.g. conflict of interest?* |
